# Onset Symptoms, Tobacco Smoking, and Progressive-Onset Phenotype Are Associated With a Delayed Onset of Multiple Sclerosis, and Marijuana Use With an Earlier Onset

**DOI:** 10.3389/fneur.2018.00418

**Published:** 2018-06-08

**Authors:** Chunrong Tao, Steve Simpson, Bruce V. Taylor, Leigh Blizzard, Robyn M. Lucas, Anne-Louise Ponsonby, Simon Broadley, AusLong/Ausimmune Investigators Group, Robyn M Lucas, Ingrid van der Mei

**Affiliations:** ^1^Menzies Institute for Medical Research, University of Tasmania, Hobart, TAS, Australia; ^2^Institute for Health & Ageing, Australian Catholic University, Melbourne, VIC, Australia; ^3^National Centre for Epidemiology and Population Health, Canberra, ACT, Australia; ^4^Murdoch Children's Research Institute, University of Melbourne, Melbourne, VIC, Australia; ^5^School of Medicine, Griffith University, Gold Coast, QLD, Australia

**Keywords:** first demyelinating event, age at symptom onset, smoking, offspring number, marijuana, multiple sclerosis

## Abstract

**Background:** Age at symptom onset (ASO) is a prognostic factor that could affect the accrual of disability in multiple sclerosis (MS) patients. Some factors are known to influence the risk of multiple sclerosis (MS), but their influence on the ASO is less well-investigated.

**Objective:** Examine the associations between known or emerging MS risk factors and ASO.

**Methods:** This was a multicenter study, incident cases (*n* = 279) with first clinical diagnosis of demyelinating event aged 18–59 years recruited at four Australian centres (latitudes 27°-43°S), from 1 November 2003 to 31 December 2006. Environmental/behavioral variables and initial symptoms were recorded at baseline interview. Linear regression was used to assess the association between risk factors and ASO.

**Results:** Five factors were significantly associated with ASO: a history of tobacco smoking was associated with 3.05-years later ASO (*p* = 0.002); a history of marijuana use was associated with 6.03-years earlier ASO (*p* < 0.001); progressive-onset cases had 5.61-years later ASO (*p* = 0.001); an initial presentation of bowel & bladder and cerebral dysfunctional were associated with 3.39 (*p* = 0.017) and 4.37-years (*p* = 0.006) later ASO, respectively. Other factors, including sex, offspring number, latitude of study site, history of infectious mononucleosis, *HLA-DR15 & HLA-A2* genotype, 25(OH)D levels, and ultraviolet radiation exposure were not associated with ASO. Including all five significant variables into one model explained 12% of the total variance in ASO.

**Conclusion:** We found a novel association between a history of tobacco smoking and later onset, whereas marijuana use was associated with earlier onset. Behavioral factors seem important drivers of MS onset timing although much of the variance remains unexplained.

## Introduction

Multiple sclerosis (MS) is a chronic inflammatory and degenerative disease of the central nervous system (CNS), caused by a complex interplay between genetic and environmental factors (1). HLA-DR15^*^01 genotype (2), lower vitamin D (3) or exposure to ultraviolet radiation (UVR) (4), tobacco use (5), and past infection with Epstein-Barr virus (6) are the main factors implicated in MS risk. Our group recently demonstrated that these risk factors could explain 63.8% of the attributable risk (7), with 53.3% of that due to the environmental factors. Other factors such as *HLA-A2* genotype (8) (protective), offspring number (9) (protective), and marijuana use (5) (detrimental), have some evidence of involvement in MS risk.

While a number of studies have examined risk factors for the onset of MS, far fewer have tested associations with age at symptom onset (ASO), which is the age at which first symptoms suggestive of future MS occur. Current studies (10, 11) mostly suggest a significant prognostic value of ASO in patients with MS, with an early onset correlated with a benign disease progression. Some studies showed that lower sun exposure in adolescence was associated with earlier ASO (12, 13). One cohort study (*n* = 895 cases) found no difference in ASO between never and ever smokers (32.29 and 32.75 years, respectively) (14). One study (*n* = 816) found that cases with *HLA-DR15* risk genotype had roughly 2.5-years earlier onset (15), but a later meta-analysis (*n* = 2,201) showed no association (16). The association between *HLA-A2* genotype and ASO has also yielded inconsistent findings (17, 18). Our group (19) found that progressive-onset patients had ~9 years later ASO than relapsing-onset patients (*p* < 0.001).

In view of the conflicting evidence presented above, this paper examines the associations between risk factors for MS onset and ASO in a cohort recruited soon after the first clinical diagnosis of CNS demyelination (FCD).

## Methods

The Ausimmune Study was an Australian multicenter case-control study (20). Case participants were aged 18–59 years and resident in a study region: Brisbane city (latitude 27° South), Newcastle city and surrounds (33°S), Geelong city and the Western Districts of Victoria (37° S), or the state of Tasmania (~41–43°S). Incident cases (*n* = 282) were referred to the study by medical specialists, following a FCD. A study neurologist confirmed the date and symptomatology of the demyelinating event(s) that led to study participation and conducted a full neurologic examination. In subsequent follow-up (AusLong study) (21), 3 cases were confirmed as non-MS, and 19 cases were confirmed as primary-progressive MS (PPMS). Among all 279 cases, 217 had been diagnosed as MS at 5-year review. Among 260 relapsing-onset cases, 219 had their first demyelinating event (FDE) during the study recruitment period (1 Nov 2003 to 31 Dec 2006), with remaining cases having had a prior, previously unrecognized neurological event.

The Ausimmune Study was approved by nine regional Human Research Ethics Committees. All participants gave written informed consent.

### Measurements

Demographic, environmental, behavioral and neurological data were collected by self-reported questionnaire and face-to-face interview. Serum samples and biometric measures were taken at face-to-face interview.

Using the Expanded Disability Status Scale (EDSS), clinical presentation was rated for seven function groups (pyramid, cerebellar, brainstem, sensory, bowel & bladder, cerebral, and visual) using 6–8 point scales. It was assessed by a neurologist via face-to-face interview at baseline. Questionnaire data collected included: a detailed smoking history of tobacco and marijuana use (ever smoked, current smoking status, age started/stopped smoking, pack-years of cigarette smoking, pack-years of marijuana use); history of infectious mononucleosis (“have you ever had glandular fever”); number and age of all live births; and for females only, the age at menarche. Participants completed a personal residence calendar (to assist recall of past events), completing for each year of life from age 6 years the location of residence and leisure time in the sun in summer and winter. With the latitude and longitude of the location, average daily ambient erythemally weighted UVR was calculated for every year of life for each participant (4). For each participant, UVR dose (kJ/m^2^) was estimated for the relevant periods of life by multiplication of the average hours per day outside in each season by the average UVR for that season, and summed over the relevant years. Annual UVR exposure was assigned by adding the summer and winter UVR dose.

Serum concentrations of 25(OH)D were measured using liquid chromatography tandem mass spectrometry. Skin reflectance on the buttock was measured using a hand-held spectrophotometer (Minolta CM-2500D) to estimate cutaneous melanin density (4). The genotyping of *HLA-DR15* (SNP rs9271366) was performed by the SNPline method (KBiosciences, Hoddesdon Herts, UK). The genotyping of *HLA-A2* (SNP rs2844821 on Illumina Custom MS Chip) was performed by the Hussman Institute for Human Genomics, University of Miami.

### Data management and analysis

ASO was calculated by subtracting the date of birth from the date of first symptom onset (as determined by review of data by the study neurologist team). Where only the year of symptom onset was recorded, the onset date was assigned as 15th of June of that year and if only the month was known it was assigned as the 15th of the month [year of FDE was recorded in 27 cases, but the results were robust when these data were omitted from the analysis (data not shown)].

Because 25(OH)D levels vary substantially by season, we deseasonalised 25(OH)D levels in each study center using a sine-cosine function, as described previously (4). The 25(OH)D level was modeled as both a continuous and categorical variable (using commonly used cut-points, <50, 50–75, >75–100, >100 nmol/L).

Due to the non-significant association between the degree of clinical presentation and ASO in patients with each clinical symptom (*p* > 0.05), clinical presentation of the seven function groups were coded as no or yes in our analysis.

Log-binomial regression was used to assess the association between categorical variables. Linear regression was used to assess the association between potential predictors and ASO. We mostly examined risk factors that occurred prior to the initial symptom onset to ensure proper temporality, except 25(OH)D was measured at the baseline review. For variables that were inherently associated with age (e.g., smoking, UVR exposure, offspring number), we summarized the information prior to a specific age (e.g., 28 years for smoking, 31 years for marijuana and offspring number) and examined the associations with ASO after this age to obtain a less biased estimate. The distribution of ASO was left skewed, and a Box-Cox power transformation was applied to reduce heteroskedasticity and thus satisfy the requirements of linear regression. All coefficients presented in the results were back-transformed to the original scale.

In preliminary analyses, participants in Tasmania had a later ASO than other study centers (mean difference: 3.19, 95% CI 0.73–5.65, *p* = 0.011). The higher ASO was likely driven by the higher average age of the Tasmanian population, but the collinearity between the population mean age and study center (*r* = 0.91, *p* < 0.001) made adjustment not possible. We therefore divided cases into five-year ASO groups (15–19 years, 20–24 years, 25–29 years, 30–34 years, 35–39 years, 40–44 years, 45–49 years, 50–54 years, 55–59 years) in each study center, and standardized the ASO to the age distribution in the whole Australian population. After standardization, ASO (from highest to lowest latitude: 37.2, 37.6, 37.0 & 37.2 years, respectively) was similar in the four different study centers.

In the basic multivariable model, we adjusted for sex, study center, and MS onset type (relapsing vs. progressive onset). For UVR exposure analysis, we did not adjust for study center due to collinearity (latitude and ambient UVR are strongly negatively correlated). We adjusted for buttock melanin density to take natural skin type into consideration. For smoking analyses, we further adjusted for tobacco or marijuana smoking, due to the significant positive correlation (*r* = 0.40, *p* < 0.001).

After the univariable and multivariable analyses of each category of exposures, we built a mutually adjusted model including all significant exposures, and examined the total variance explained by these exposures. The relative contribution of each variable was calculated as the change of the residual sum of squares when comparing the model without the variable with the full model.

We conducted a sensitivity analysis restricted to cases with FDE during the recruitment period and excluding those whose ASO was less certain. We performed another separate analysis restricted to cases who had converted to MS by a 5-year follow-up, (21) thus excluding cases who may never convert to MS. We were concerned that some associations were the result of an age or cohort effect. To examine this we used the controls (*n* = 545) and assigned the same ASO of the cases to their age and sex-matched controls. With the Australian Electoral Roll, we randomly selected controls and matched to cases on age (within two years), sex, and study region (60% participated of those originally contacted). The final case:control matching ratio averaged 1:2 (20). If an association was also observed in controls, then that is likely due to an age or cohort effect.

All statistical analyses were undertaken using Stata/SE 12.1 (College Park, TX).

## Results

The mean ASO for the total cohort was 37.3 years and 76.7% were females. ASO for those who had converted to MS at 5-year follow-up (*n* = 217) was 37.0 years and for cases with FDE during the recruitment period (*n* = 219) was 36.9 years. All primary analyses were based on all patients with FDE (*n* = 279), and sensitivity analyses including patients had converted to MS at 5-year review (*n* = 217) or patients with FDE during recruitment period (*n* = 219) were presented in the last section of result.

### Sex, HLA-DR15, HLA-A2 and history of infectious mononucleosis

We found no significant associations between ASO and sex, history of IM, *HLA-DR15* genotype, or *HLA-A2* genotype (Table 1; Supplementary Table 1).

**Table 1:** Associations between sex, IM history, HLA-DR15 genotype, HLA-A2 genotype, 25(OH)D, UVR exposure and age of symptom onset.

		**Univariable**		**Multivariable**	
	**No. (%)**	β **(95% CI)**	***p***	β **(95% CI)**	***p***
**SEX^a^**					
Male	65 (23.30)	37.50 (35.17, 39.83) ^c^		37.21 (34.90, 39.52)^c^	
Female	214 (76.70)	−0.28 (−2.94, 2.38)	0.84	+0.15 (-2.49, 2.80)	0.91
**PAST HISTORY OF IM^a^**					
No	200 (72.99)	37.61 (36.30, 38.92)^c^		37.7 (36.41, 39.00)^c^	
Yes	74 (27.01)	−1.18 (−3.70, 1.34)	0.36	−1.33 (−3.83, 1.18)	0.30
**PAST HISTORY OF IM (EXCLUDING THOSE WHO DID NOT KNOW WHETHER THEY HAD A HISTORY OF IM)^a^**					
No	181 (70.98)	37.23 (35.85, 38.61)^c^		37.32 (35.96, 38.67)^c^	
Yes	74 (29.02)	−0.85 (−3.41, 1.71)	0.51	−0.96 (−3.49, 1.58)	0.46
**HLA-DR15 (RS9271366)^a^**					
AA	106 (43.80)	37.19 (35.38, 39.00)^c^		36.89 (34.91, 38.86)^c^	
AG	123 (50.83)	+0.40 (−2.07, 2.86)	0.75	+1.42 (−1.26, 4.09)	0.30
GG	13 (5.37)	+3.18 (−2.26, 8.63)	0.25	+4.74 (−2.43, 11.91)	0.20
Trend			0.38		0.15
**HLA–A2 (RS2844821)^a^**					
AA	117 (54.42)	36.93 (35.22, 38.64)^c^		37.17 (35.43, 38.92)^c^	
AG	98 (45.58)	+1.79 (−0.74, 4.31)	0.17	+1.38 (−1.24, 4.01)	0.30
**SEASON OF FDE^b^**					
Spring	70 (25.09)	36.9 (34.47, 39.34)^c^		37.2 (34.69, 39.7)^c^	
Summer	72 (25.81)	+1.00 (−2.27, 4.26)	0.55	0.01 (−3.39, 3.42)	0.99
Autumn	61 (21.86)	+0.05 (−3.28, 3.37)	0.98	−0.42 (−3.8, 2.97)	0.81
Winter	76 (27.24)	+0.40 (−2.87, 3.66)	0.81	0.43 (−2.89, 3.76)	0.80
Deseasonalised 25(OH)D, continuous (per 10 nmol/L increase)^b^		−0.04 (−0.48, 0.40)	0.87	−0.03 (−0.48, 0.41)	0.88
**DESEASONALISED 25(OH)D^b^**					
<50 nmol/L	33 (15.57)	36.95 (33.68, 40.22)^c^		36.92 (33.64, 40.19)^c^	
50–75 nmol/L	70 (33.02)	−0.19 (−4.16, 3.77)	0.92	−0.33 (−4.32, 3.65)	0.87
76 – 100 nmol/L	70 (33.02)	−0.68 (−4.65, 3.28)	0.74	−0.75 (−4.72, 3.22)	0.71
> 100 nmol/L	39 (18.40)	+1.26 (−3.18, 5.71)	0.58	+1.19 (−3.25, 5.64)	0.60
Trend			0.67		0.67
**WINTER UVR DOSE IN THE 15 YEARS BEFORE ONSET^b^**					
0–0.93 x10^2^ kJ/m^2^	64 (24.81)	39.62 (37.43, 41.81)^c^		39.68 (37.47, 41.9)^c^	
> 0.93–1.59 × 10^2^ kJ/m^2^	65 (25.19)	−3.05 (−6.12, 0.02)	0.05	–***3.37 (***–***6.46***, –***0.28)***	***0.032***
> 1.59–2.51 × 10^2^ kJ/m^2^	64 (24.81)	−1.95 (−5.04, 1.14)	0.22	−2.06 (−5.22, 1.09)	0.20
> 2.51–8.47 × 10^2^ kJ/m^2^	65 (25.19)	−1.43 (−4.51, 1.65)	0.36	−1.37 (−4.50, 1.76)	0.39
Trend			0.53		0.57
**SUMMER UVR DOSE IN THE 15 YEARS BEFORE ONSET^b^**					
0–5.53 x10^2^ kJ/m^2^	64 (24.71)	39.82 (37.66, 41.97)^c^		39.54 (37.35, 41.73)^c^	
> 5.53–7.37 × 10^2^ kJ/m^2^	65 (25.10)	–***3.91 (***–***6.91***, –***0.91)***	***0.011***	–***3.75 (***–***6.77***, –***0.73)***	***0.015***
> 7.37–9.99 × 10^2^ kJ/m^2^	65 (25.10)	–***3.95 (**−**6.95***, –***0.95)***	***0.010***	–***3.87 (***–***6.93***, –***0.81)***	***0.013***
> 9.99–18.99 × 10^2^ kJ/m^2^	65 (25.10)	+0.38 (−2.66, 3.42)	0.81	+1.07 (−2.15, 4.28)	0.52
Trend			0.81		0.65
**ANNUAL UVR DOSE IN THE 15 YEARS BEFORE ONSET^b^**					
0**–**6.87 × 10^2^ kJ/m^2^	64 (24.81)	39.35 (37.18, 41.52)^c^		39.09 (36.89, 41.3)^c^	
> 6.87–9.30 × 10^2^ kJ/m^2^	65 (25.19)	–***3.13 (***–***6.15***, –***0.10)***	***0.043***	−3.01 (−6.07, 0.04)	0.053
> 9.30–12.00 × 10^2^ kJ/m^2^	64 (24.81)	–***3.42 (***–***6.45***, –***0.38)***	***0.027***	–***3.26 (***–***6.37***, –***0.15)***	***0.040***
> 12.00–22.46 × 10^2^ kJ/m^2^	65 (25.19)	+0.85 (−2.21, 3.91)	0.59	+1.41 (−1.79, 4.61)	0.39
Trend			0.64		0.48

*Statistical significance (p < 0.05) is denoted in bold and italics*.

a *adjusted for sex, MS onset type and study center*;

b *adjusted for sex, MS onset type and buttock melanin density*;

c *mean ASO of the reference group*.

*MS, multiple sclerosis; IM, infectious mononucleosis; UVR, ultraviolet radiation; FDE, first demyelinating event*.

### 25(OH)D and UVR dose

No seasonal pattern was seen in the association between season of FDE and ASO (*F* = 0.61, *p* = 0.72). There was no association between deseasonalised 25(OH)D level and ASO (Table 1). Time between FDE and 25(OH)D being taken did not modify the association between 25(OH)D and ASO (p_interactio_ *n* = 0.38). Cumulative UVR dose (summer, winter, and combined) within different risk windows (e.g., 5, 10, and 15 years) prior to onset and UVR dose during adolescence (6-15 years) showed no convincing associations with ASO (Table 1; Supplementary Table 2).

### Onset type and initial symptoms

ASO of progressive-onset patients was 5.61-years later than relapsing-onset patients (*p* = 0.013) (Table 2). There was no association between pyramidal, brainstem, sensory, visual dysfunction and ASO. Cases with bowel/bladder and cerebral symptoms had 3.49-years (*p* = 0.017) and 4.37-years (*p* = 0.006) later onset, respectively, persisting on restriction to relapsing-onset cases. Patients with cerebellar function impairment had a 2.52-years later onset than those without cerebellar function impairment (*p* = 0.057), and this association became significant when restricting to relapsing-onset cases.

**Table 2:** Associations between onset type and initial symptomatology and age of symptom onset.

		**Univariable analysis**		**Multivariable analysis^a^**	
	**No. (%)**	**β (95% CI)**	***p***	**β (95% CI)**	***p***
**MS ONSET TYPE**					
Relapsing-onset	258 (92.47)	36.99 (35.84, 38.15)^b^		36.95 (35.8, 38.09)^b^	
Progressive-onset	21 (7.53)	*+**4.86 (0.44, 9.28)***	***0.031***	*+**5.61 (1.17, 10.05)***	***0.013***
**ONSET SYMPTOMS**					
Pyramidal dysfunction -no	150 (58.37)	37.51 (35.98, 39.04)^b^		37.86 (36.33, 39.38)^b^	
Pyramidal dysfunction -yes	107 (41.63)	+0.21 (−2.17, 2.58)	0.86	−0.52 (−2.94, 1.90)	0.67
Cerebellar dysfunction -no	185 (72.27)	36.89 (35.53, 38.26)^b^		37.01 (35.66, 38.37)^b^	
Cerebellar dysfunction -yes	71 (27.73)	*+**2.83 (0.24, 5.41)***	***0.032***	+2.52 (−0.08, 5.12)	0.06
Brainstem dysfunction -no	192 (75.59)	37.43 (36.06, 38.79)^b^		37.4 (36.06, 38.75)^b^	
Brainstem dysfunction -yes	62 (24.41)	−0.13 (−2.90, 2.64)	0.93	0.15 (−2.59, 2.89)	0.92
Sensory dysfunction -no	114 (45.6)	37.6 (35.83, 39.37)^b^		37.56 (35.81, 39.3)^b^	
Sensory dysfunction -yes	136 (54.4)	−0.48 (−2.88, 1.92)	0.70	−0.33 (−2.70, 2.05)	0.79
Bowel & Bladder dysfunction -no	210 (79.55)	36.84 (35.56, 38.12)^b^		36.85 (35.58, 38.12)^b^	
Bowel & Bladder dysfunction -yes	54 (20.45)	*+**3.39 (0.57, 6.20)***	***0.019***	*+**3.49 (0.63, 6.34)***	***0.017***
Cerebral dysfunction -no	218 (83.52)	36.71 (35.47, 37.95)^b^		36.87 (35.64, 38.1)^b^	
Cerebral dysfunction-yes	43 (16.48)	*+**5.12 (2.08, 8.15)***	***0.001***	*+**4.37 (1.28, 7.46)***	***0.006***
Visual dysfunction -no	178 (70.92)	37.64 (36.25, 39.03)^b^		37.74 (36.38, 39.10)^b^	
Visual dysfunction -yes	73 (29.08)	−0.80 (−3.38, 1.77)	0.54	−1.00 (−3.54, 1.54)	0.44
**ONSET SYMPTOMS IN RELAPSING-ONSET CASES**					
Pyramidal dysfunction -no	147 (61.51)	37.22 (35.70, 38.75)^b^		37.35 (35.84, 38.86)^b^	
Pyramidal dysfunction -yes	92 (38.49)	−0.09 (−2.55, 2.37)	0.94	−0.36 (−2.80, 2.09)	0.77
Cerebellar dysfunction -no	179 (74.27)	36.55 (35.18, 37.92)^b^		36.55 (35.19, 37.91)^b^	
Cerebellar dysfunction -yes	62 (25.73)	*+**2.82 (0.11, 5.52)***	***0.041***	*+**2.91 (0.21, 5.60)***	***0.034***
Brainstem dysfunction -no	179 (74.9)	36.79 (35.39, 38.18)^b^		36.78 (35.41, 38.16)^b^	
Brainstem dysfunction -yes	60 (25.1)	+1.06 (−1.72, 3.83)	0.46	+1.14 (−1.61, 3.90)	0.42
Sensory dysfunction -no	106 (45.3)	37.15 (35.34, 38.96)^b^		37.12 (35.33, 38.91)^b^	
Sensory dysfunction -yes	128 (54.7)	−0.29 (−2.73, 2.15)	0.81	−0.21 (−2.63, 2.21)	0.87
Bowel & Bladder dysfunction -no	201 (81.71)	36.52 (35.23, 37.81)^b^		36.43 (35.16, 37.71)^b^	
Bowel & Bladder dysfunction -yes	45 (18.29)	*+**3.33 (0.31, 6.34)***	***0.031***	*+**3.90 (0.88, 6.93)***	***0.011***
Cerebral dysfunction -no	205 (84.36)	36.35 (35.10, 37.61)^b^		36.47 (35.21, 37.72)^b^	
Cerebral dysfunction -yes	38 (15.64)	*+**5.20 (2.02, 8.39)***	***0.001***	*+**4.54 (1.30, 7.78)***	***0.006***
Visual dysfunction -no	166 (70.34)	37.10 (35.68, 38.51)^b^		37.23 (35.83, 38.63)^b^	
Visual dysfunction -yes	70 (29.66)	−0.24 (−2.84, 2.37)	0.86	−0.63 (−3.22, 1.96)	0.63

*Statistical significance (p < 0.05) is denoted in bold and italics*.

a *adjusted for sex, MS type, and study center*;

b *mean ASO of the reference group*.

### Smoking of tobacco and marijuana

Ever smokers had ~5.11-years later onset than never smokers (*p* < 0.001) (Table 3). The magnitudes were similar between past smokers and current smokers. Cases with an earlier ASO might have had less opportunity to start smoking, so we tested this by only including cases with ASO ≥ 28 years (as 27 was the oldest age of taking up smoking). While attenuating the magnitude, a history of smoking remained significantly associated with ASO (Table 3). We repeated the analysis in the matched controls, and no difference of ASO was shown (never smokers vs. ever smokers: adjusted β = +0.11 years, 95% CI −1.54–1.77, *p* = 0.89), suggesting the significant association in cases was not due to an age or cohort effect. Among ever smokers, the ASO was similar whether the smoking was taken up early (<16 years) or later (p_interactio_ *n* = 0.26), or the duration was longer or shorter (p_interactio_ *n* = 0.94). Among cases with ASO ≥ 28 years, total pack years of tobacco use before age 27 was not associated with ASO.

**Table 3:** Associations between smoking behaviors and age of symptom onset.

		**Univariable**		**Multivariable^a^**	
	**No. (%)**	**β (95% CI)**	***P***	**β (95% CI)**	***P***
**SMOKING EVER**					
No	103 (37.59)	35.6 (33.79, 37.41)^b^		34.24 (32.41, 36.06)^b^	
Yes	171 (62.41)	*+**2.71 (0.42, 5.00)***	***0.021***	*+**5.11 (2.74, 7.48)***	***<0.001***
**SMOKING EVER (ASO ≥ 28 YEARS)**					
No	79 (35.59)	38.71 (37.2, 40.22)^b^		37.89 (36.45, 39.34)^b^	
Yes	143 (64.41)	+1.79 (−0.12, 3.71)	0.07	*+**3.05 (1.16, 4.95)***	***0.002***
**SMOKING STATUS**					
Never smoked	103 (37.59)	35.6 (33.78, 37.41)^b^		34.21 (32.38, 36.03)^b^	
Past smokers	101 (36.86)	*+**2.62 (0.04, 5.20)***	***0.047***	+4.80 (2.21, 7.38)	***<0.001***
Current smokers	70 (25.55)	+2.85 (−0.01, 5.70)	0.05	+5.66 (2.69, 8.64)	***<0.001***
Trend			***0.037***		***<0.001***
**SMOKING STATUS (ASO ≥ 28 YEARS)**					
Never smoked	79 (35.59)	38.71 (37.2, 40.22)^b^		37.89 (36.44, 39.34)^b^	
Past smokers	83 (37.39)	+2.12 (−0.05, 4.30)	0.06	*+**3.05 (0.95, 5.14)***	***0.004***
Current smokers	60 (27.03)	+1.34 (−1.01, 3.68)	0.27	*+**3.06 (0.65, 5.47)***	***0.013***
Trend			0.22		***0.010***
**AGE UPTAKE SMOKING (ASO ≥ 28 YEARS)**					
≥16 years	84 (58.74)	41.45 (39.85, 43.05)^b^		41.21 (39.72, 42.7)^b^	
<16 years	59 (41.26)	−1.77 (−4.22, 0.68)	0.16	−1.35 (−3.66, 0.97)	0.26
**DURATION OF SMOKING BEFORE AGE 27 (ASO ≥ 28 YEARS)**					
≤10 years	55 (40.44)	40.97 (38.99, 42.95)^b^		40.58 (38.67, 42.5)^b^	
>10 years	81 (59.56)	−0.51 (−3.07, 2.04)	0.69	+0.10 (−2.40, 2.60)	0.94
**TOTAL PACK YEARS OF SMOKING BEFORE AGE 27 (ASO ≥ 28 YEARS)**					
0	93 (41.33)	39.19 (37.77, 40.61)		39.10 (37.71, 40.49)	
1–2000	58 (25.78)	+0.50 (−1.81, 2.81)	0.67	+0.61 (−1.67, 2.89)	0.60
>2000–20000	74 (32.89)	+2.05 (−0.15, 4.25)	0.07	+2.24 (0.07, 4.41)	0.043
Trend			0.07		0.048
**MARIJUANA EVER**					
No	190 (69.6)	38.58 (37.26, 39.9)^b^		39.23 (37.91, 40.55)^b^	
Yes	83 (30.4)	–***4.04 (***–***6.43***, –***1.64)***	***0.001***	−6.03 (−8.62, −3.45)	***<0.001***
**MARIJUANA EVER (ASO ≥ 31 YEARS)**					
No	151 (74.02)	41.29 (40.24, 42.34)^b^		41.5 (40.43, 42.57)^b^	
Yes	53 (25.98)	–***2.20 (***–***4.14***, –***0.27)***	***0.026***	–***2.80 (***–***4.89***, –***0.71)***	***0.009***
**MARIJUANA STATUS**					
Never	190 (69.6)	38.58 (37.26, 39.90)^b^		38.56 (37.25, 39.87)^b^	
Past users	63 (23.08)	–***4.08 (***–***6.73***, –***1.43)***	***0.003***	–***3.98 (***–***6.63***, –***1.34)***	***0.003***
Current users	20 (7.33)	−3.92 (−8.20, 0.37)	0.07	−3.46 (−7.87, 0.94)	0.12
Trend			***0.003***		***0.004***
**MARIJUANA STATUS (ASO ≥ 31 YEARS)**					
Never	151 (74.02)	41.28 (40.24, 42.33)^b^		41.25 (40.22, 42.28)^b^	
Past users	39 (19.12)	−1.50 (−3.71, 0.70)	0.18	−1.35 (−3.58, 0.88)	0.24
Current users	14 (6.86)	–***4.06 (***–***7.16***, –***0.95)***	***0.010***	–***3.88 (***–***7.09***, –***0.66)***	***0.018***
Trend			***0.008***		***0.016***
**TOTAL PACK YEARS OF MARIJUANA USE BEFORE AGE 30 (ASO ≥ 31 YEARS)**					
0	180 (86.96)	40.96 (40.01, 41.92)		40.93 (39.99, 41.87)	
1–30	13 (6.28)	+1.13 (−2.70, 4.96)	0.56	+1.48 (−2.34, 5.30)	0.45
>30	14 (6.76)	−3.16 (−6.34, 0.02)	0.052	−3.07 (−6.29, 0.14)	0.06
Trend			0.14		0.19

*Statistical significance (p < 0.05) was denoted in bold and italics*.

a *adjusted for sex, MS type, study centers and tobacco smoking status/marijuana use status*;

b *mean ASO of the reference group*.

Marijuana use was more common in male cases than in females (OR = 0.50, *p* = 0.020), and a history of marijuana use was positively associated with smoking (OR = 8.05, *p* < 0.001). A history of marijuana use was associated with a 6.03-years earlier ASO (Table 3). The magnitudes were similar between past users and current users. We next evaluated associations among cases with ASO ≥ 31 years (30 years was the oldest age of taking up marijuana in this cohort). Magnitudes were attenuated but still significant in this subgroup (Table 3). Among cases with ASO ≥ 31 years, total pack years of marijuana use before age 30 was not associated with ASO.

### Offspring numbers and age at menarche

Having children was strongly associated with a later ASO (Table 4). The magnitude was potentially biased since those with a later ASO may have had different opportunities to have children. We therefore chose a cut-off point that balanced sample size against the offspring number information, and by restricting to those with ASO ≥ 31 years, we could include 204 cases and 56.9% of participant offspring. A significant dose-dependent association remained, albeit with reduced magnitude. This dose-dependent association was found in both male and female participants (p_interactio_ *n* = 0.11). When we repeated the analyses in the controls, having more children was also significantly associated with a later ASO (0 vs. 1: β = 4.55, *p* < 0.001; 0 vs. ≥2: β = 10.41, *p* < 0.001; p_trend_ < 0.001), and restricting to those with ASO ≥ 31 years still showed a significant association (0 vs. 1: β = 1.39, *p* = 0.16; 0 vs. ≥ 2: β = 3.48, *p* < 0.001; p_trend_ < 0.001). This suggests that the significant association of offspring number and ASO in cases was largely driven by an age or cohort effect.

**Table 4:** Associations between offspring numbers/age at menarche and age of symptom onset.

		**Univariable**		**Multivariable**	
	**No. (%)**	**β (95% CI)**	***p***	**β (95% CI)**	***p***
**OFFSPRING NUMBER^a^**					
0	111 (39.78)	31.16 (29.7, 32.63)^c^		31.37 (29.9, 32.83)^c^	
1	39 (13.98)	*+**5.97 (3.01, 8.92)***	***<0.001***	*+**5.51 (2.57, 8.44)***	***<0.001***
2 or more	129 (46.24)	*+**11.12 (9.06, 13.18)***	***<0.001***	*+**10.88 (8.84, 12.93)***	***<0.001***
Trend			***<0.001***		***<0.001***
**OFFSPRING NUMBER (ASO ≥ 31 YEARS)^b^**					
0	89 (43.00)	39.25 (37.99, 40.5)^c^		39.22 (37.99, 40.45)^c^	
1	37 (17.87)	+1.67 (−0.74, 4.09)	0.18	+1.39 (−0.98, 3.77)	0.25
2 or more	81 (39.13)	*+**3.42 (1.47, 5.36)***	***0.001***	*+**3.61 (1.69, 5.54)***	***<0.001***
Trend			***0.001***		***<0.001***
**AGE OF MENARCHE^a^**					
8–12 years	98 (46.23)	37.07 (35.17, 38.97)^c^		37.13 (35.24, 39.01)^c^	
13–14 years	87 (41.04)	+1.13 (−1.64, 3.9)	0.43	+0.98 (−1.8, 3.75)	0.49
15–23 years	27 (12.74)	–***4.19 (***–***8.24***, –***0.15)***	***0.042***	−3.72 (−7.76, 0.32)	0.07
Trend			0.22		0.27

*Statistical significance (p < 0.05) is denoted in bold and italics*.

a *Models adjusted for sex, MS type, and study centre*;

b *samples limited to those with ASO ≥ age 31 years and summarizing offspring number < age 31 years, models adjusted for sex, MS type, and study centers*.

c *mean ASO of the reference group*.

The age of the participant when having their first child (≤20, 21–25, 26–30, 31–35, ≥36) was not associated with ASO; the average ASO was around 41 years for each child birth age group. There was no association between age of menarche and ASO (*p* = 0.27).

### Mutually adjusted model

We next built a combined model based on the significant findings (tobacco smoking, marijuana use, MS onset type, cerebral dysfunction, and bowel & bladder dysfunction). The final model explained ~12% of the total variance in ASO, with marijuana use and tobacco smoking having the largest relative contributions (Table 5).

**Table 5:** Mutually adjusted model including all factors associated with age of symptom onset.

	**All cases (adjusted *R*^2^ = 0.12)**		
	**β (95% CI)**	***P***	**RC**
Smoking status			5.0%
Never smoking	35.14 (33.31, 36.98)^a^		
Past users	+3.80 (1.21, 6.40)	***0.004***	
Current users	+4.72 (1.74, 7.71)	***0.002***	
Trend		***0.001***	
Marijuana history			9.2%
Never marijuana	39.47 (38.17, 40.78)^a^		
Past users	−5.49 (−8.29, −2.70)	***<0.001***	
Current users	−7.23 (−11.75, −2.71)	***0.002***	
Trend		***<0.001***	
MS onset type			0.6%
RRMS	37.58 (36.46, 38.69)^a^		
PPMS	+2.23 (−2.05, 6.52)	0.31	
Cerebral function			5.9%
No	36.96 (35.78, 38.14)^a^		
Yes	+4.58 (1.68, 7.48)	***0.002***	
Bowel & bladder function			1.2%
No	37.3 (36.09, 38.51)^a^		
Yes	+2.13 (−0.61, 4.87)	0.13	

*Statistical significance (p < 0.05) is denoted in bold and italics. RC, relative contribution*.

a *mean ASO of the reference group*.

### Sensitivity analyses

Sensitivity analyses that restricted to cases with FDE during the study recruitment period or diagnosed MS cases did not materially alter the results (Supplementary Tables).

## Discussion

In this comprehensive study, we systematically evaluated the association between ASO and risk factors for MS onset. A model including all the significant factors explained 12% of the total variance. Of the individual risk factors, a history of tobacco smoking, progressive-onset type, and cerebral or bowel and bladder symptoms at FDE were associated with a later onset, while marijuana use was associated with an earlier onset. *HLA-A2* genotype, *HLA-DR15* genotype, latitude of study site, past UVR dose, current 25(OH)D levels, history of IM, offspring number, age of menarche, and sex were not associated with ASO.

A strength of our study is the detailed information on exposure variables, as well as the study neurologist interview and review of medical records for optimal assessment of the ASO. Also, our findings showed a robustness when repeating the analysis among those diagnosed with MS by 5-year review (excluding some cases who may not convert to MS), as well as restricting to cases with FDE during the study recruitment period (excluding some whose ASO was slightly less certain). A challenge was that some exposures were intrinsically associated with ASO simply as a function of time. So we assessed the temporal relationship through analyzing some exposure variables before some age points and analyzing the association with ASO after this point. This ensured that the participants included in the analysis had an equal opportunity of exposure, but it substantially reduced the power and assumed that there was nothing unusual about those with a later vs. earlier onset. To examine whether some observed associations were due to an age or cohort effect, we tested whether an effect existed in the age and sex matched controls. Based on that analysis, we believe that the association between offspring number and ASO was not a true association.

In agreement with previous studies (19, 22, 23), progressive-onset patients showed a later ASO than relapsing-onset patients. In this study, patients with cerebral dysfunction (related to cognition and mood) or bowel and bladder symptoms at FDE had a 4.4 and 3.5 years later ASO, respectively, and cerebellar function impairment (related to ataxia and coordination) was significantly associated with a later onset in relapse-onset patients (2.91 years later ASO). A previous study (24) found no association between psychiatric (*p* = 0.31) or cognitive (*p* = 0.93) symptoms and ASO, but a significant relationship between sphincteric symptoms and ASO (*p* = 0.012). Marked differences in the ASO between that study and presented study (~30 years vs. 37 years) may account for the divergent findings.

The finding that tobacco smoking delayed the average ASO by 4 years is counterintuitive given that it is a risk factor for MS (5). Two other studies have shown a similar directions of effect, with magnitudes of 2.60 years (*n* = 7,883) (25) and 0.82 years (*n* = 540) (13). A null association between tobacco smoking and ASO in healthy controls supported that this association was not due to an age or cohort effect. However, we note that our controls did not show the expected decline in ever smoker status by birth cohort 2004–05 as the general Australian population (26). Some hidden confounder or responder bias, e.g., with older controls being more health conscious and less likely to be smokers may account for this (26). However, the control sample was still a preferable comparison source than the general Australian population, since controls resided in the same regional locations as the cases and were age/sex-matched. The participation rate for controls was lower than cases (20) but this potential volunteer bias would be unlikely to lead to altered patterns of smoking by age among the controls only. A good explanation for the opposite relationship for tobacco use in MS onset (27) versus ASO (13, 25) is challenging. It could relate to the anti-inflammatory effects of nicotine upon T-cells, B-cells, and even dendritic cells (28). Tobacco smoking may have a greater impact on neurodegeneration rather than inflammation and immune dysfunction (29) and older onset cases may have a greater neurodegenerative component (30).

Marijuana use was associated with a 6.0-years earlier ASO, despite the Ausimmune Study (5) showing no association between marijuana use and MS risk [never vs. ever, adjusted OR 0.91 (0.84–1.54)]. Other research has demonstrated a detrimental effect of marijuana on the CNS. Two studies (31, 32) have showed that marijuana use was associated with poorer cognitive performance in MS patients. Thus, there is some evidence supporting potential deleterious impacts of marijuana use in MS, though the means by which this occurs, requires further study.

Consistent with previous research (12, 13, 17, 33), we found no association between sex, history of infectious mononucleosis, *HLA-A2* or *HLA-DR15* genotype and ASO. Latitude of study site, deseasonalised 25(OH)D levels and UVR dose were not associated with ASO in the present study. Using the large MSBase global dataset (*n* = 22,162), we previously showed an inverse association between latitude and ASO (every 10° increase in latitude was associated with a 0.82-year earlier onset, *p* = 2.20 × 10^−13^) (19). However, in line with the present study, subgroup analysis restricting to Australian participants showed a non-significant association. Our previous study also found that UVR exposure from age 6 to 15 years and lifetime actinic damage were not associated with ASO (34). Another cross-sectional study (12) (*n* = 1,161) found that vitamin D-associated SNPs and vitamin D supplementation (multivitamin/vitamin D or fatty fish) were not associated with ASO.

While we identified some factors that were associated with ASO, we explained only 12% of the total variance, thus leaving the majority unexplained. We demonstrated that marijuana use was associated with an earlier MS onset and that a history of tobacco smoking was associated with a later ASO. Further research is required to better understand these opposite effects and underlying pathways. These results, if corroborated and supported in other studies, may aid in the understanding of MS and potentially contribute to delaying MS onset.

## Author contributions

CT analyzed the data, wrote the draft manuscript and completed revisions. IvdM designed this study and formulated the hypotheses for this analysis. All authors contributed to data interpretation, critically revised the manuscript for important intellectual content, and read and approved the final manuscript.

### Conflict of interest statement

The authors declare that the research was conducted in the absence of any commercial or financial relationships that could be construed as a potential conflict of interest.
