# Supplementary material for: Onset Symptoms, Tobacco Smoking, and Progressive-Onset Phenotype Are Associated With a Delayed Onset of Multiple Sclerosis, and Marijuana Use With an Earlier Onset
Source: Front Neurol. 2018 Jun 8;9:418. doi: 10.3389/fneur.2018.00418 (PMC6003245; doi:10.3389/fneur.2018.00418)
Supplement: Supplementary file 1 [file Data_Sheet_1.docx]

Updated Supplementary Tables

Table 1 Sensitivity analysis of sex, history of infectious mononucleosis (IM), *HLA-DR15* genotype, *HLA-A2* genotype, for the total cohort in the Ausimmune Study, and restricting to cases with FDE during the recruitment period, and those with diagnosed MS by 5-year review

|  | | Primary analysis | | Restricted to classic FDE | | Restricted to MS | |
| --- | --- | --- | --- | --- | --- | --- | --- |
|  | | **β (95% CI)** | **p** | **β (95% CI)** | **p** | **β (95% CI)** | **p** |
| Sex | |  |  |  |  |  |  |
|  | Male | 37.21 (34.90, 39.52) ^a^ |  | 37.16 (34.58, 39.74) ^a^ |  | 37.00 (34.08, 39.93) ^a^ |  |
|  | Female | +0.15 (-2.49, 2.80) | 0.91 | -0.52 (-3.48, 2.43) | 0.73 | -0.03 (-3.29, 3.24) | 0.99 |
| Past history of IM | |  |  |  |  |  |  |
|  | No | 37.7 (36.41, 39.00) ^a^ |  | 37.03 (35.60, 38.46) ^a^ |  | 37.59 (36.09, 39.08) ^a^ |  |
|  | Yes | -1.33 (-3.83, 1.18) | 0.30 | -1.07 (-3.85, 1.71) | 0.45 | -1.91 (-4.80, 0.98) | 0.19 |
| *HLA-DR15 (rs9271366)* | |  |  |  |  |  |  |
|  | AA | 36.89 (34.91, 38.86) ^a^ |  | 36.06 (33.93, 38.20) ^a^ |  | 36.99 (34.60, 39.38) ^a^ |  |
|  | AG | +1.42 (-1.26, 4.0 9) | 0.30 | +2.11 (-0.82, 5.03) | 0.16 | +0.53 (-2.65, 3.72) | 0.74 |
|  | GG | +4.74 (-2.43, 11.91) | 0.20 | +3.14 (-4.59, 10.87) | 0.43 | +4.55 (-2.94, 12.05) | 0.23 |
|  | Trend |  | 0.15 |  | 0.14 |  | 0.38 |
| *HLA-A2 (rs2844821)* | |  |  |  |  |  |  |
|  | AA | 37.17 (35.43, 38.92) ^a^ |  | 36.69 (34.78, 38.59) ^a^ |  | 36.84 (34.85, 38.83) ^a^ |  |
|  | AG | +1.38 (-1.24, 4.01) | 0.30 | +1.32 (-1.57, 4.20) | 0.37 | +1.52 (-1.55, 4.59) | 0.33 |

Statistical significance (p<0.05) is denoted in bold and italics. Models are adjusted for sex, MS type, and study centre a: mean ASO of the reference group

Table 2 Sensitivity analysis of UVR exposure of the total cohort in the Ausimmune Study by restricting to cases with FDE during recruitment period and those with diagnosed MS before 5-year review

|  | | Primary analysis | | Restricted to classic FDE | | Restricted to MS | |
| --- | --- | --- | --- | --- | --- | --- | --- |
|  | | **β (95% CI)** | **p** | **β (95% CI)** | **p** | **β (95% CI)** | **p** |
| Winter UV dose within  5 years before onset ^b^ | |  |  |  |  |  |  |
|  | 0-0.27 x10^2^ kJ/m^2^ | 38.06 (35.73, 40.40) ^a^ |  | 38.07 (35.5, 40.64) ^a^ |  | 37.92 (35.44, 40.41) ^a^ |  |
|  | >0.27-0.45 x10^2^ kJ/m^2^ | -1.33 (-4.54, 1.88) | 0.42 | -2.00 (-5.54, 1.53) | 0.27 | -2.89 (-6.49, 0.72) | 0.12 |
|  | >0.45-0.75 x10^2^ kJ/m^2^ | -2.16 (-5.44, 1.13) | 0.20 | -1.68 (-5.30, 1.94) | 0.36 | -2.62 (-6.26, 1.03) | 0.16 |
|  | >0.75-3.16 x10^2^ kJ/m^2^ | +0.17 (-3.13, 3.46) | 0.92 | -1.51 (-5.20, 2.18) | 0.42 | +0.86 (-2.80, 4.52) | 0.65 |
|  | Trend |  | 0.97 |  | 0.48 |  | 0.72 |
| Winter UV dose within  10 years before onset ^b^ | |  |  |  |  |  |  |
|  | 0-0.58 x10^2^ kJ/m^2^ | 38.36 (36.08, 40.64) ^a^ |  | 38.76 (36.2, 41.31) ^a^ |  | 38.21 (35.76, 40.65) ^a^ |  |
|  | >0.58 - 0.95x10^2^ kJ/m^2^ | -2.07 (-5.29, 1.14) | 0.21 | -3.16 (-6.7, 0.38) | 0.08 | -3.42 (-7.05, 0.21) | 0.07 |
|  | >0.95 - 1.59x10^2^ kJ/m^2^ | -2.21 (-5.45, 1.03) | 0.18 | -2.62 (-6.24, 0.99) | 0.16 | -2.84 (-6.40, 0.72) | 0.12 |
|  | >1.59 - 5.26 x10^2^ kJ/m^2^ | -0.28 (-3.51, 2.96) | 0.87 | -2.18 (-5.82, 1.46) | 0.24 | +0.38 (-3.25, 4.01) | 0.84 |
|  | Trend |  | 0.85 |  | 0.31 |  | 0.92 |
| Winter UV dose within  15 years before onset ^b^ | |  |  |  |  |  |  |
|  | 0-0.93 x10^2^ kJ/m^2^ | 38.95 (36.73, 41.17) ^a^ |  | 39.07 (36.6, 41.53) ^a^ |  | 38.88 (36.48, 41.27) ^a^ |  |
|  | >0.93 - 1.59x10^2^ kJ/m^2^ | -2.15 (-5.25, 0.96) | 0.18 | -1.86 (-5.31, 1.58) | 0.29 | -3.79 (-7.21, -0.36) | ***0.03*** |
|  | >1.59 - 2.51x10^2^ kJ/m^2^ | -2.31 (-5.47, 0.85) | 0.15 | -2.95 (-6.39, 0.48) | 0.09 | -2.66 (-6.27, 0.94) | 0.15 |
|  | >2.51 - 8.47x10^2^ kJ/m^2^ | -0.64 (-3.78, 2.50) | 0.69 | -2.29 (-5.78, 1.2) | 0.20 | +0.14 (-3.35, 3.64) | 0.94 |
|  | Trend |  | 0.69 |  | 0.15 |  | 0.85 |
| Summer UV dose within  5 years before onset ^b^ | |  |  |  |  |  |  |
|  | 0-0.45 x10^2^ kJ/m^2^ | 37.08 (34.79, 39.38) ^a^ |  | 36.54 (33.85, 39.22) ^a^ |  | 36.93 (34.49, 39.37) ^a^ |  |
|  | >0.45 - 0.62x10^2^ kJ/m^2^ | -0.69 (-3.90, 2.52) | 0.68 | -0.34 (-4.01, 3.33) | 0.86 | -0.66 (-4.18, 2.86) | 0.71 |
|  | >0.62 - 0.72x10^2^ kJ/m^2^ | -1.13 (-4.36, 2.09) | 0.49 | -1.30 (-4.90, 2.30) | 0.48 | -2.13 (-5.7, 1.45) | 0.24 |
|  | >0.72 - 0.95x10^2^ kJ/m^2^ | 2.48 (-0.86, 5.83) | 0.15 | +2.49 (-1.32, 6.30) | 0.20 | 2.83 (-0.98, 6.63) | 0.15 |
|  | Trend |  | 0.21 |  | 0.30 |  | 0.37 |
| Summer UV dose within  10 years before onset ^b^ | |  |  |  |  |  |  |
|  | 0-3.28 x10^2^ kJ/m^2^ | 38.40 (36.13, 40.67) ^a^ |  | 37.82 (35.18, 40.46) ^a^ |  | 38.60 (36.17, 41.03) ^a^ |  |
|  | >3.28 - 4.92x10^2^ kJ/m^2^ | -3.27 (-6.44, -0.11) | 0.043 | -3.17 (-6.74, 0.39) | 0.08 | ***-3.88 (-7.35, -0.41)*** | ***0.028*** |
|  | >4.92 - 6.80x10^2^ kJ/m^2^ | -2.66 (-5.84, 0.52) | 0.10 | -2.72 (-6.34, 0.89) | 0.14 | ***-4.13 (-7.57, -0.68)*** | ***0.019*** |
|  | >6.80 - 9.53x10^2^ kJ/m^2^ | +1.36 (-1.97, 4.69) | 0.42 | +1.59 (-2.17, 5.35) | 0.41 | +1.80 (-2.04, 5.64) | 0.36 |
|  | Trend |  | 0.46 |  | 0.38 |  | 0.76 |
| Summer UV dose within  15 years before onset ^b^ | |  |  |  |  |  |  |
|  | 0-5.53 x10^2^ kJ/m^2^ | 38.92 (36.73, 41.10) ^a^ |  | 38.49 (35.91, 41.07) ^a^ |  | 39.49 (37.16, 41.82) ^a^ |  |
|  | >5.53 - 7.37x10^2^ kJ/m^2^ | ***-3.49 (-6.51, -0.46)*** | ***0.024*** | ***-3.53 (-6.91, -0.16)*** | ***0.040*** | ***-4.40 (-7.72, -1.08)*** | ***0.009*** |
|  | >7.37 - 9.99x10^2^ kJ/m^2^ | ***-3.68 (-6.71, -0.65)*** | ***0.017*** | ***-3.43 (-6.86, 0.01)*** | ***0.050*** | ***-5.35 (-8.62, -2.09)*** | ***0.001*** |
|  | >9.99 - 18.99x10^2^ kJ/m^2^ | +2.28 (-0.95, 5.50) | 0.17 | +2.12 (-1.57, 5.80) | 0.26 | +2.45 (-1.25, 6.16) | 0.19 |
|  | Trend |  | 0.29 |  | 0.26 |  | 0.73 |
| UV dose within 5 years  before onset ^b^ | |  |  |  |  |  |  |
|  | 0-2.02 x10^2^ kJ/m^2^ | 37.07 (34.77, 39.37) ^a^ |  | 36.36 (33.70, 39.01) ^a^ |  | 36.89 (34.43, 39.34) ^a^ |  |
|  | >2.02 - 3.07x10^2^ kJ/m^2^ | -0.95 (-4.17, 2.27) | 0.56 | -0.33 (-3.92, 3.27) | 0.86 | -1.25 (-4.83, 2.33) | 0.50 |
|  | >3.07 - 4.03x10^2^ kJ/m^2^ | -0.20 (-3.44, 3.04) | 0.90 | +0.18 (-3.50, 3.85) | 0.93 | -1.15 (-4.76, 2.45) | 0.53 |
|  | >4.03 - 7.82x10^2^ kJ/m^2^ | +1.82 (-1.54, 5.18) | 0.29 | +1.73 (-2.11, 5.56) | 0.38 | +2.14 (-1.63, 5.92) | 0.27 |
|  | Trend |  | 0.27 |  | 0.36 |  | 0.36 |
| UV dose within 10 years  before onset ^b^ | |  |  |  |  |  |  |
|  | 0-4.38 x10^2^ kJ/m^2^ | 37.74 (35.41, 40.08) ^a^ |  | 37.04 (34.30, 39.78) ^a^ |  | 37.61 (35.05, 40.16) ^a^ |  |
|  | >4.38 - 6.07x10^2^ kJ/m^2^ | -2.49 (-5.68, 0.71) | 0.13 | -1.96 (-5.55, 1.63) | 0.28 | -2.56 (-6.10, 0.97) | 0.16 |
|  | >6.07 - 8.02x10^2^ kJ/m^2^ | -0.92 (-4.19, 2.34) | 0.58 | -0.38 (-4.11, 3.35) | 0.84 | -1.95 (-5.59, 1.70) | 0.30 |
|  | >8.02 - 14.50x10^2^ kJ/m^2^ | +1.49 (-1.87, 4.85) | 0.39 | +1.39 (-2.48, 5.27) | 0.48 | +1.76 (-2.09, 5.62) | 0.37 |
|  | Trend |  | 0.27 |  | 0.32 |  | 0.43 |
| UV dose within 15 years  before onset ^b^ | |  |  |  |  |  |  |
|  | 0-6.87 x10^2^ kJ/m^2^ | 39.06 (36.86, 41.26) ^a^ |  | 38.70 (36.19, 41.20) ^a^ |  | 38.94 (36.54, 41.34) ^a^ |  |
|  | >6.87 - 9.30x10^2^ kJ/m^2^ | ***-3.92 (-6.95, -0.90)*** | ***0.011*** | ***-3.99 (-7.32, -0.66)*** | ***0.019*** | ***-3.51 (-6.90, -0.13)*** | ***0.042*** |
|  | >9.30 - 12.00x10^2^ kJ/m^2^ | ***-3.18 (-6.32, -0.05)*** | ***0.047*** | ***-3.41 (-6.80, -0.02)*** | ***0.049*** | ***-4.14 (-7.67, -0.61)*** | ***0.021*** |
|  | >12.00 - 22.46x10^2^ kJ/m^2^ | +1.39 (-1.80, 4.57) | 0.39 | +1.80 (-1.84, 5.44) | 0.33 | +1.26 (-2.37, 4.90) | 0.50 |
|  | Trend |  | 0.37 |  | 0.38 |  | 0.74 |
| Winter UV dose between  6-15 ^b^ | |  |  |  |  |  |  |
|  | 0-0.71x10^2^ kJ/m^2^ | 37.83 (35.53, 40.13) ^a^ |  | 37.53 (34.94, 40.12) ^a^ |  | 36.91 (34.33, 39.49) ^a^ |  |
|  | >0.71-1.12 x10^2^ kJ/m^2^ | +0.06 (-3.19, 3.30) | 0.97 | +0.20 (-3.43, 3.82) | 0.92 | +1.20 (-2.50, 4.91) | 0.52 |
|  | >1.12-2.26 x10^2^ kJ/m^2^ | -1.13 (-4.36, 2.11) | 0.49 | -1.19 (-4.79, 2.40) | 0.52 | -1.05 (-4.79, 2.69) | 0.58 |
|  | >2.26-7.68 x10^2^ kJ/m^2^ | -1.21 (-4.48, 2.05) | 0.47 | -1.92 (-5.54, 1.70) | 0.30 | -0.57 (-4.22, 3.09) | 0.76 |
|  | Trend |  | 0.36 |  | 0.22 |  | 0.52 |
| Summer UV dose between  6-15 ^b^ | |  |  |  |  |  |  |
|  | 0-4.53 x10^2^ kJ/m^2^ | 37.19 (34.9, 39.48) ^a^ |  | 35.81 (33.27, 38.36) ^a^ |  | 37.44 (34.97, 39.91) ^a^ |  |
|  | >4.53-6.27 x10^2^ kJ/m^2^ | -0.42 (-3.64, 2.80) | 0.80 | +0.83 (-2.77, 4.44) | 0.65 | -2.18 (-5.79, 1.44) | 0.24 |
|  | >6.27-7.23 x10^2^ kJ/m^2^ | +1.49 (-1.73, 4.70) | 0.36 | +2.79 (-0.72, 6.29) | 0.12 | +0.49 (-3.12, 4.10) | 0.79 |
|  | >7.23-7.99 x10^2^ kJ/m^2^ | -0.83 (-4.13, 2.47) | 0.62 | +0.06 (-3.63, 3.76) | 0.97 | -0.98 (-4.75, 2.79) | 0.61 |
|  | Trend |  | 0.96 |  | 0.63 |  | 0.96 |
| Annual UV dose between  6-15 ^b^ | |  |  |  |  |  |  |
|  | 0-5.64x10^2^ kJ/m^2^ | 37.18 (34.88, 39.47) ^a^ |  | 36.51 (33.92, 39.10) ^a^ |  | 37.16 (34.64, 39.68) ^a^ |  |
|  | >5.64-7.39 x10^2^ kJ/m^2^ | +0.40 (-2.83, 3.63) | 0.81 | +0.50 (-3.10, 4.10) | 0.79 | -0.58 (-4.18, 3.03) | 0.75 |
|  | >7.39-9.19 x10^2^ kJ/m^2^ | -0.39 (-3.63, 2.85) | 0.81 | +0.14 (-3.48, 3.77) | 0.94 | -0.94 (-4.68, 2.81) | 0.62 |
|  | >9.19-10.87 x10^2^ kJ/m^2^ | +0.27 (-3.03, 3.56) | 0.87 | +0.33 (-3.34, 4.00) | 0.86 | +0.05 (-3.68, 3.78) | 0.98 |
|  | Trend |  | 1.00 |  | 0.91 |  | 0.95 |

Statistical significance (p<0.05) is denoted in bold and italics. Models adjusted for sex, MS type, and buttock melanin density a: mean ASO of the reference group

Table 3 Sensitivity analysis of MS type and initial clinical symptomatology for the total cohort in the Ausimmune Study, and restricting to cases with FDE during recruitment period and those with diagnosed MS by 5-year review

|  | | Multivariable analysis | | Restricted to classic FDE | | Restricted to MS | |
| --- | --- | --- | --- | --- | --- | --- | --- |
|  | | **β (95% CI)** | **p** | **β (95% CI)** | **p** | **β (95% CI)** | **p** |
| MS onset type | |  |  |  |  |  |  |
|  | Relapsing-onset | 36.95 (35.8, 38.09) ^b^ |  |  |  | 36.52 (35.18, 37.86) ^b^ |  |
|  | Progressive-onset | ***+5.61 (1.17, 10.05)*** | ***0.013*** |  |  | ***+5.91 (1.34, 10.47)*** | ***0.011*** |
| Onset symptoms | |  |  |  |  |  |  |
|  | Pyramid dysfunction-no | 37.86 (36.33, 39.38) ^b^ |  | 37.15 (35.53, 38.77) ^b^ |  | 37.82 (35.98, 39.67) ^b^ |  |
|  | Pyramid dysfunction-yes | -0.52 (-2.94, 1.90) | 0.67 | -0.15 (-2.83, 2.52) | 0.91 | -0.89 (-3.66, 1.88) | 0.53 |
|  | Cerebellar dysfunction-no | 37.01 (35.66, 38.37) ^b^ |  | 36.38 (34.93, 37.83) ^b^ |  | 36.86 (35.23, 38.48) ^b^ |  |
|  | Cerebellar dysfunction-yes | +2.52 (-0.08, 5.12) | 0.057 | ***+3.45 (0.44, 6.45)*** | ***0.025*** | +1.99 (-0.91, 4.88) | 0.18 |
|  | Brainstem dysfunction-no | 37.4 (36.06, 38.75) ^b^ |  | 36.88 (35.38, 38.37) ^b^ |  | 37.28 (35.69, 38.87) ^b^ |  |
|  | Brainstem dysfunction-yes | 0.15 (-2.59, 2.89) | 0.92 | +0.16 (-2.86, 3.19) | 0.92 | -0.57 (-3.73, 2.60) | 0.73 |
|  | Sensory dysfunction-no | 37.56 (35.81, 39.3) ^b^ |  | 36.78 (34.89, 38.68) ^b^ |  | 37.91 (35.85, 39.96) ^b^ |  |
|  | Sensory dysfunction-yes | -0.33 (-2.70, 2.05) | 0.79 | +0.12 (-2.52, 2.75) | 0.93 | -1.35 (-4.12, 1.43) | 0.34 |
|  | Bowel & Bladder dysfunction-no | 36.85 (35.58, 38.12) ^b^ |  | 36.36 (34.98, 37.75) ^b^ |  | 36.52 (35.02, 38.02) ^b^ |  |
|  | Bowel & Bladder dysfunction-yes | ***+3.49 (0.63, 6.34)*** | ***0.017*** | ***+3.62 (0.33, 6.91)*** | ***0.031*** | ***+3.59 (0.27, 6.91)*** | ***0.034*** |
|  | Cerebral dysfunction-no | 36.87 (35.64, 38.1) ^b^ |  | 36.39 (35.03, 37.76) ^b^ |  | 36.6 (35.16, 38.04) ^b^ |  |
|  | Cerebral dysfunction-yes | ***+4.37 (1.28, 7.46)*** | ***0.006*** | ***+4.19 (0.69, 7.69)*** | ***0.019*** | ***+4.58 (1.10, 8.06)*** | ***0.010*** |
|  | Visual dysfunction-no | 37.74 (36.38, 39.10) ^b^ |  | 37.16 (35.62, 38.69) ^b^ |  | 37.41 (35.85, 38.98) ^b^ |  |
|  | Visual dysfunction-yes | -1.00 (-3.54, 1.54) | 0.44 | -0.61 (-3.37, 2.14) | 0.66 | -1.11 (-4.22, 2.00) | 0.49 |
| Onset symptoms in  relapsing-onset cases | |  |  |  |  |  |  |
|  | Pyramid dysfunction-no | 37.35 (35.84, 38.86) ^b^ |  |  |  | 37.11 (35.27, 38.95) ^b^ |  |
|  | Pyramid dysfunction-yes | -0.36 (-2.80, 2.09) | 0.77 |  |  | -0.65 (-3.47, 2.17) | 0.65 |
|  | Cerebellar dysfunction-no | 36.55 (35.19, 37.91) ^b^ |  |  |  | 36.21 (34.56, 37.85) ^b^ |  |
|  | Cerebellar dysfunction-yes | ***+2.91 (0.21, 5.60)*** | ***0.034*** |  |  | +2.42 (-0.61, 5.45) | 0.12 |
|  | Brainstem dysfunction-no | 36.78 (35.41, 38.16) ^b^ |  |  |  | 36.47 (34.82, 38.11) ^b^ |  |
|  | Brainstem dysfunction-yes | +1.14 (-1.61, 3.90) | 0.42 |  |  | +0.62 (-2.58, 3.81) | 0.71 |
|  | Sensory dysfunction-no | 37.12 (35.33, 38.91) ^b^ |  |  |  | 37.37 (35.23, 39.5) ^b^ |  |
|  | Sensory dysfunction-yes | -0.21 (-2.63, 2.21) | 0.87 |  |  | -1.27 (-4.14, 1.59) | 0.38 |
|  | Bowel & Bladder dysfunction-no | 36.43 (35.16, 37.71) ^b^ |  |  |  | 35.93 (34.41, 37.45) ^b^ |  |
|  | Bowel & Bladder dysfunction-yes | ***+3.90 (0.88, 6.93)*** | ***0.011*** |  |  | ***+4.24 (0.62, 7.86)*** | ***0.022*** |
|  | Cerebral dysfunction-no | 36.47 (35.21, 37.72) ^b^ |  |  |  | 36.05 (34.56, 37.53) ^b^ |  |
|  | Cerebral dysfunction-yes | ***+4.54 (1.30, 7.78)*** | ***0.006*** |  |  | ***+4.89 (1.20, 8.59)*** | ***0.009*** |
|  | Visual dysfunction-no | 37.23 (35.83, 38.63) ^b^ |  |  |  | 36.73 (35.1, 38.37) ^b^ |  |
|  | Visual dysfunction-yes | -0.63 (-3.22, 1.96) | 0.63 |  |  | -0.65 (-3.86, 2.56) | 0.69 |

Statistical significance (p<0.05) was denoted in bold and italics. a: adjusted for sex, MS type, and study centres; b: mean ASO of the reference group

Table 4 Sensitivity analysis of smoking behaviours for the total cohort in the Ausimmune Study, and restricting to cases with FDE during the recruitment period and those with diagnosed MS by 5-year review

|  | | Primary analysis | | Restricted to classic FDE | | Restricted to MS | |
| --- | --- | --- | --- | --- | --- | --- | --- |
|  | | β (95% CI) | P |  |  | β (95% CI) | P |
| Smoking ever ^a^ | |  |  |  |  |  |  |
|  | No | 34.24 (32.41, 36.06) ^c^ |  | 33.48 (31.48, 35.48) ^c^ |  | 34.62 (32.46, 36.78) ^c^ |  |
|  | Yes | ***+5.11 (2.74, 7.48)*** | ***<0.001*** | ***+5.42 (2.80, 8.04)*** | ***<0.001*** | ***+3.99 (1.17, 6.82)*** | ***0.005*** |
| Smoking ever ^b^ | |  |  |  |  |  |  |
|  | No | 36.73 (35.22, 38.25) ^c^ |  | 37.1 (35.54, 38.66) ^c^ |  | 39.1 (37.34, 40.86) ^c^ |  |
|  | Yes | ***+3.96 (1.95, 5.96)*** | ***<0.001*** | ***+3.21 (1.14, 5.28)*** | ***0.002*** | +1.63 (-0.63, 3.90) | 0.16 |
| Smoking status ^a^ | |  |  |  |  |  |  |
|  | Never smoked | 34.65 (32.82, 36.49) ^c^ |  | 33.44 (31.44, 35.45) ^c^ |  | 34.61 (32.44, 36.77) ^c^ |  |
|  | Past smokers | ***+3.96 (1.36, 6.56)*** | ***0.003*** | ***+4.80 (1.91, 7.69)*** | ***0.001*** | ***+3.84 (0.77, 6.90)*** | ***0.014*** |
|  | Current smokers | ***+4.92 (1.9, 7.94)*** | ***0.001*** | ***+6.36 (3.14, 9.58)*** | ***<0.001*** | ***+4.28 (0.73, 7.84)*** | ***0.018*** |
|  | Trend |  | ***0.001*** |  | ***<0.001*** |  | ***0.013*** |
| Smoking status ^b^ | |  |  |  |  |  |  |
|  | Never smoked | 36.99 (35.45, 38.52) ^c^ |  | 37.09 (35.53, 38.64) ^c^ |  | 39.13 (37.37, 40.89) ^c^ |  |
|  | Past smokers | ***+3.66 (1.41, 5.90)*** | ***0.001*** | ***+2.85 (0.52, 5.17)*** | ***0.016*** | +1.94 (-0.54, 4.42) | 0.13 |
|  | Current smokers | ***+3.24 (0.69, 5.79)*** | ***0.013*** | ***+3.73 (1.14, 6.31)*** | ***0.005*** | +1.09 (-1.75, 3.93) | 0.45 |
|  | Trend |  | ***0.010*** |  | ***0.005*** |  | ***0.392*** |
| Age of smoking begin ^b^ | |  |  |  |  |  |  |
|  | ≥16 | ***+4.39 (2.17, 6.61)*** | ***<0.001*** | 39.82 (38.18, 41.46) ^c^ |  | 40.92 (39.24, 42.59) ^c^ |  |
|  | <16 | ***+3.26 (0.75, 5.76)*** | ***0.011*** | +0.22 (-2.36, 2.81) | 0.87 | -1.17 (-3.81, 1.48) | 0.39 |
| Duration of smoking before 28 ^b^ | |  |  |  |  |  |  |
|  | ≤ 10 years | ***+2.34 (0.11, 4.57)*** | ***0.039*** | 39.64 (37.47, 41.82) ^c^ |  | 40.12 (37.96, 42.29) ^c^ |  |
|  | >10 | +2.46 (-0.11, 5.03) | 0.06 | +0.37 (-2.42, 3.16) | 0.80 | +0.73 (-2.09, 3.55) | 0.61 |
| Marijuana ever ^a^ | |  |  |  |  |  |  |
|  | No | 39.23 (37.91, 40.55) ^c^ |  | 38.72 (37.27, 40.17) ^c^ |  | 38.63 (37.11, 40.15) ^c^ |  |
|  | Yes | ***-6.03 (-8.62, -3.45)*** | ***<0.001*** | ***-5.79 (-8.52, -3.07)*** | ***<0.001*** | ***-5.25 (-8.33, -2.18)*** | ***0.001*** |
| Marijuana status ^a^ | |  |  |  |  |  |  |
|  | Never | 38.56 (37.25, 39.87) ^c^ |  | 38.74 (37.28, 40.19) ^c^ |  | 38.62 (37.09, 40.14) ^c^ |  |
|  | Past users | ***-3.98 (-6.63, -1.34)*** | ***0.003*** | ***-5.58 (-8.51, -2.65)*** | ***<0.001*** | ***-5.43 (-8.74, -2.12)*** | ***0.001*** |
|  | Current users | -3.46 (-7.87, 0.94) | 0.12 | ***-6.55 (-11.18, -1.92)*** | ***0.006*** | -4.58 (-10.09, 0.93) | 0.10 |
|  | Trend |  | ***0.004*** |  | ***<0.001*** |  | ***0.003*** |
| Marijuana ever after 31 | |  |  |  |  |  |  |
|  | No | 41.5 (40.43, 42.57) ^c^ |  | 41.07 (39.9, 42.24) ^c^ |  | 41.51 (40.32, 42.70) ^c^ |  |
|  | Yes | ***-2.80 (-4.89, -0.71)*** | ***0.009*** | ***-3.33 (-5.46, -1.21)*** | ***0.002*** | -1.95 (-4.44, 0.53) | 0.12 |
| Marijuana status after 31 | |  |  |  |  |  |  |
|  | Never | 41.25 (40.22, 42.28) ^c^ |  | 41.08 (39.91, 42.25) ^c^ |  | 41.49 (40.31, 42.68) ^c^ |  |
|  | Past users | -1.35 (-3.58, 0.88) | 0.24 | ***-2.89 (-5.24, -0.54)*** | ***0.016*** | -1.09 (-3.87, 1.68) | 0.44 |
|  | Current users | ***-3.88 (-7.09, -0.66)*** | ***0.018*** | ***-4.57 (-7.80, -1.34)*** | ***0.006*** | ***-4.30 (-8.14, -0.47)*** | ***0.028*** |
|  | Trend |  | ***0.016*** |  | ***0.001*** |  | ***0.041*** |

Statistical significance (p<0.05) is denoted in bold and italics. a: adjusted for sex, MS type, study centre, and tobacco smoking status/marijuana use status; b:excluding those with ASO<28 years old, adjusted for sex, MS type, study centre, and tobacco smoking status/marijuana use status. c: mean ASO of the reference group

Table 5 Sensitivity analysis of offspring number and age at menarche for the total cohort in the Ausimmune Study, and restricting to participants with FDE during the recruitment period and those with diagnosed MS before 5-year review

|  | | Primary analysis | | Restricted to classic FDE | | Restricted to MS | |
| --- | --- | --- | --- | --- | --- | --- | --- |
| Offspring number ^a^ | | β (95% CI) | p | β (95% CI) | p | β (95% CI) | p |
|  | 0 | 31.37 (29.9, 32.83) ^d^ |  | 31.57 (29.95, 33.19) ^d^ |  | 31.07 (29.37, 32.77) ^d^ |  |
|  | 1 | ***+5.51 (2.57, 8.44)*** | ***<0.001*** | ***+4.29 (1.01, 7.56)*** | ***0.010*** | +6.39 (3.06, 9.71) | ***<0.001*** |
|  | 2 or more | ***+10.88 (8.84, 12.93)*** | ***<0.001*** | +10.06 (7.74, 12.38) | ***<0.001*** | +10.99 (8.58, 13.39) | ***<0.001*** |
|  | Trend |  | ***<0.001*** |  | ***<0.001*** |  | ***<0.001*** |
| Offspring number ^b^ | |  |  |  |  |  |  |
|  | 0 | 39.22 (37.99, 40.45) ^d^ |  | 38.85 (37.54, 40.15) ^d^ |  | 40.05 (38.59, 41.51) ^d^ |  |
|  | 1 | +1.39 (-0.98, 3.77) | 0.25 | +0.66 (-1.91, 3.22) | 0.62 | +1.51 (-1.36, 4.38) | 0.30 |
|  | 2 or more | ***+3.61 (1.69, 5.54)*** | ***<0.001*** | ***+3.52 (1.37, 5.67)*** | ***0.001*** | +2.17 (-0.05, 4.39) | 0.06 |
|  | Trend |  | ***<0.001*** |  | ***0.002*** |  | 0.06 |
| Age of menarche ^c^ | |  |  |  |  |  |  |
|  | 8-14 | 37.60 (36.24, 38.96) ^d^ |  | 37.14 (35.60, 38.68) ^d^ |  | 37.44 (35.92, 38.97) ^d^ |  |
|  | 15-23 | ***-4.16 (-8.00, -0.33)*** | ***0.033*** | ***-5.95 (-10.09, -1.81)*** | ***0.005*** | ***-5.03 (-9.46, -0.60)*** | ***0.026*** |

Statistical significance (p<0.05) is denoted in bold and italics. a: Models adjusted for sex, MS type, study centre; b: restricted to those with ASO greater than 31 and summarizing offspring number before age 31, adjusted for sex, MS type, study centres; c: adjusted for MS type, study centre d: mean ASO of the reference group
